# Supplementary material for: Genome-Wide Association Study for Body Conformation Traits and Fitness in Czech Holsteins
Source: Animals (Basel). 2022 Dec 13;12(24):3522. doi: 10.3390/ani12243522 (PMC10375906; doi:10.3390/ani12243522)
Supplement: Supplementary file 1 [file animals-12-03522-s001.zip › animals-1966423-supplementary.pdf]

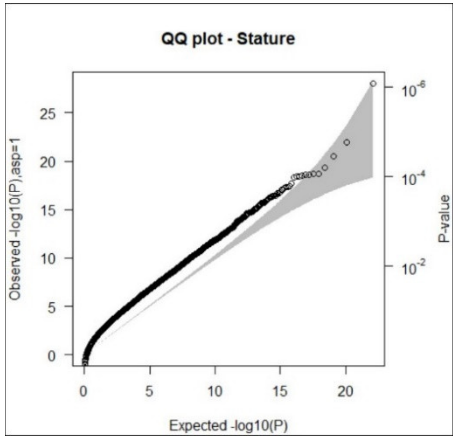

λ = 1.81

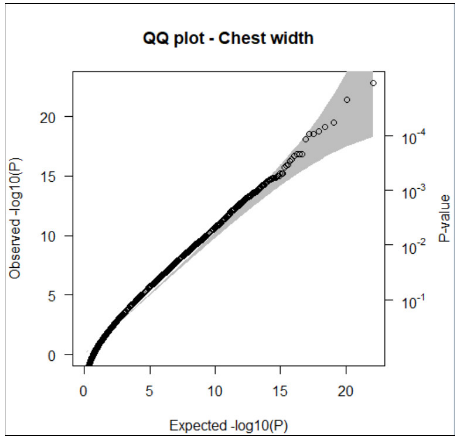

λ =-0.64

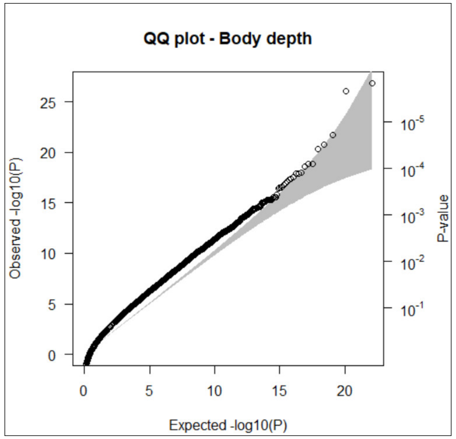

λ = 0.59

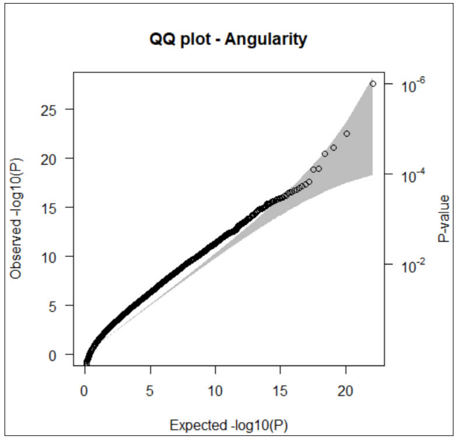

λ = 0.82

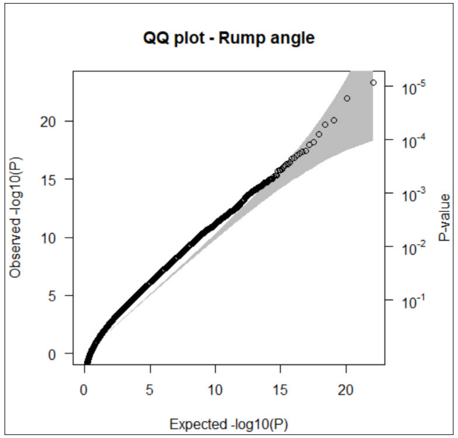

λ = 0.37

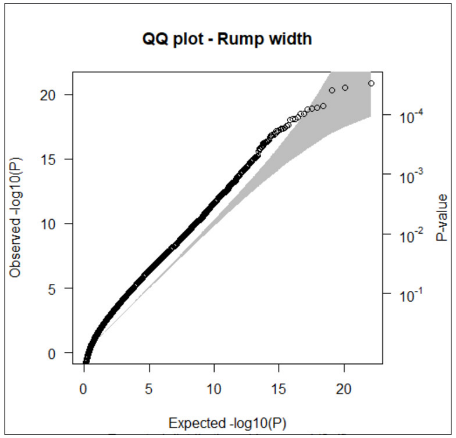

λ = 0.82

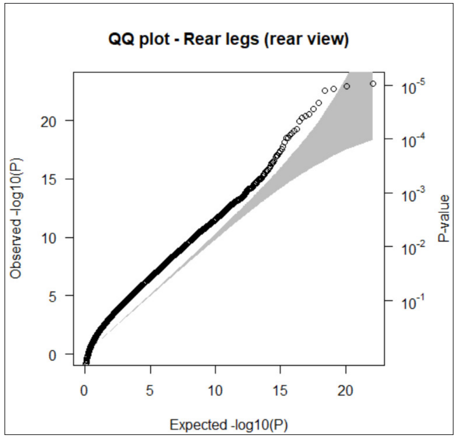

λ = 1.41

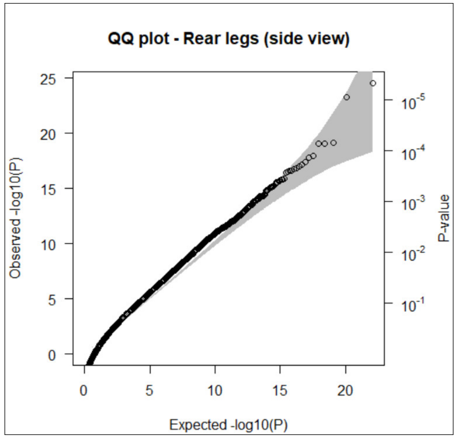

λ = -0.81

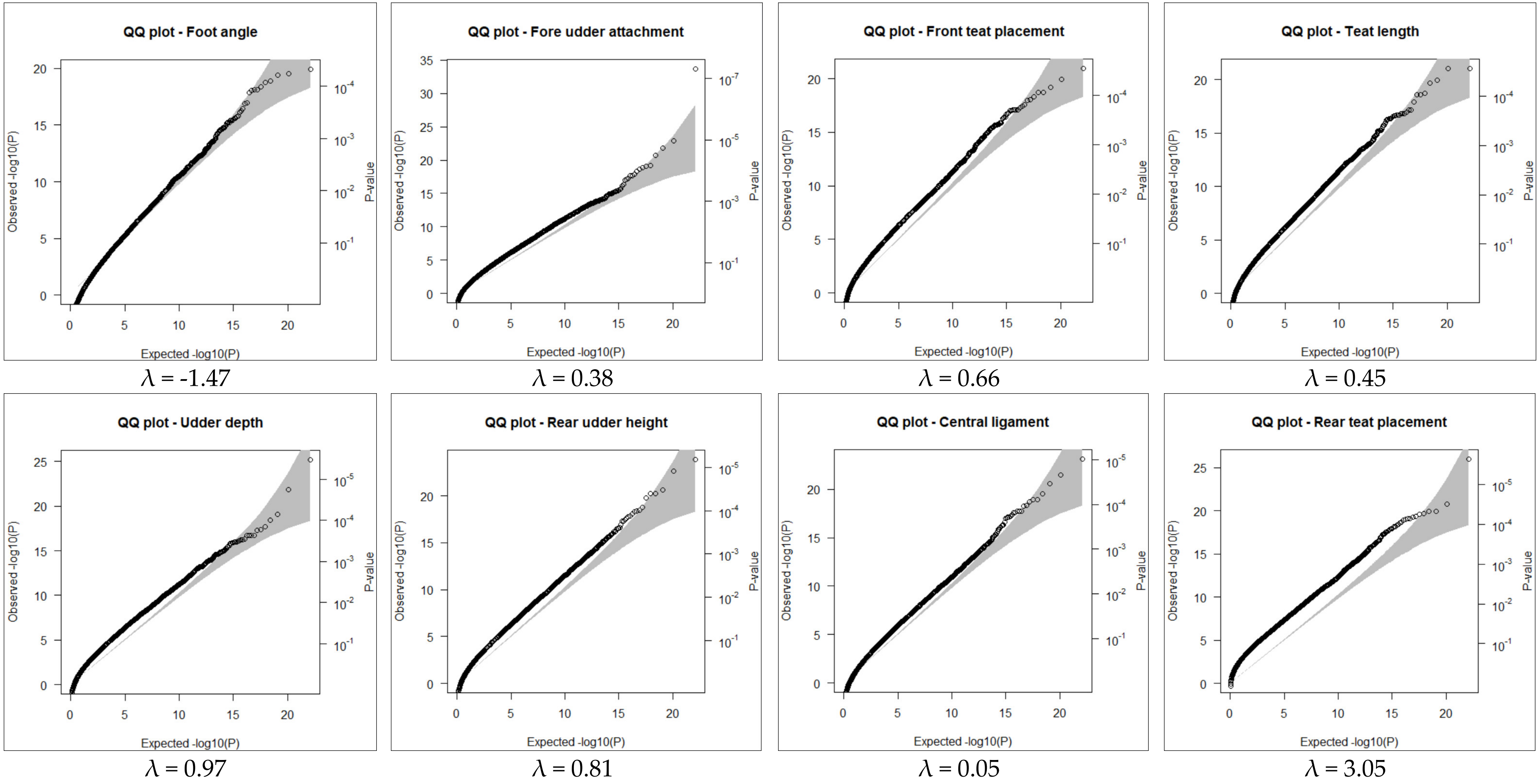
QQ plot - Foot angle
λ = -1.47
QQ plot - Fore udder attachment
λ = 0.38
QQ plot - Front teat placement
λ = 0.66
QQ plot - Teat length
λ = 0.45
QQ plot - Udder depth
λ = 0.97
QQ plot - Rear udder height
λ = 0.81
QQ plot - Central ligament
λ = 0.05
QQ plot - Rear teat placement
λ = 3.05
Observed -log10(P)
Expected -log10(P)
P-value

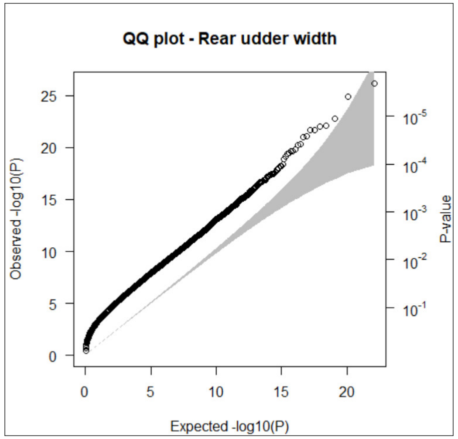

$\lambda = 4.10$

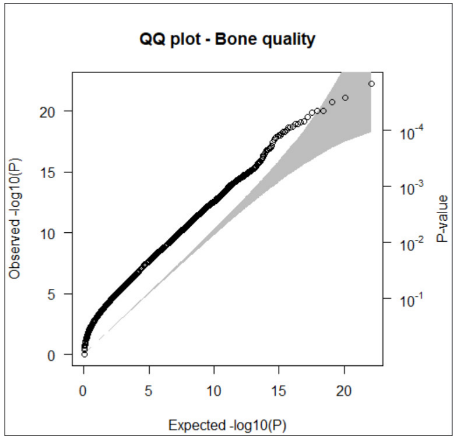

$\lambda = 3.64$

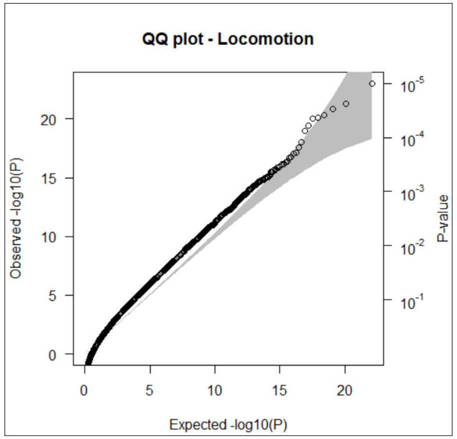

$\lambda = 0.01$

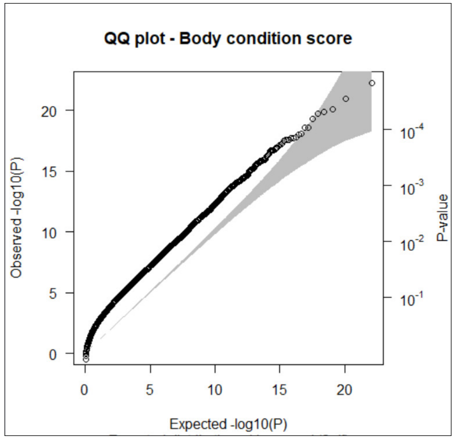

$\lambda = 2.80$

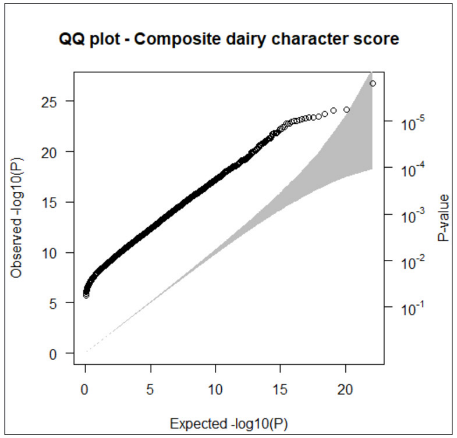

$\lambda = 12.08$

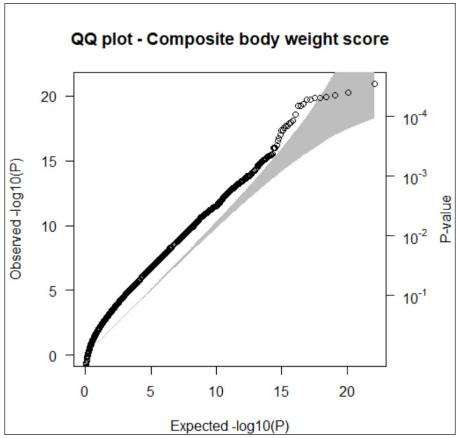

$\lambda = 1.75$

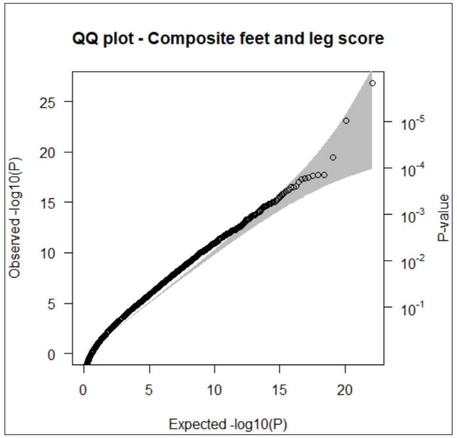

$\lambda = -0.18$

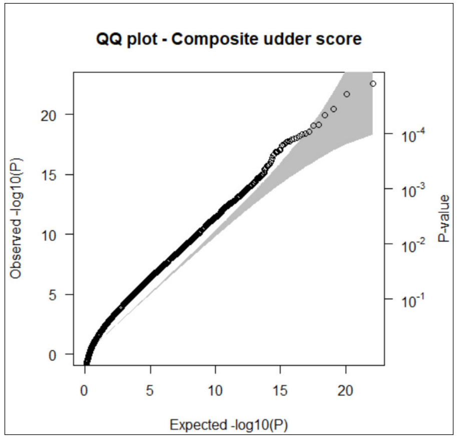

$\lambda = 0.95$

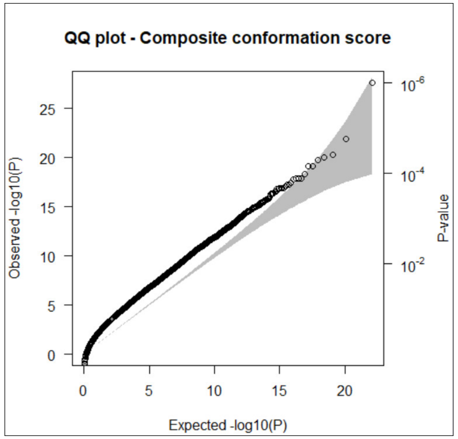

$\lambda = 1.80$

Figure S1. Quantile-quantile (Q-Q) plots with the 95% confidence interval (grey colored area) and genomic inflation factor (below the QQ plots) of the GWAS analysis for 25 linear type traits in Czech Holstein.

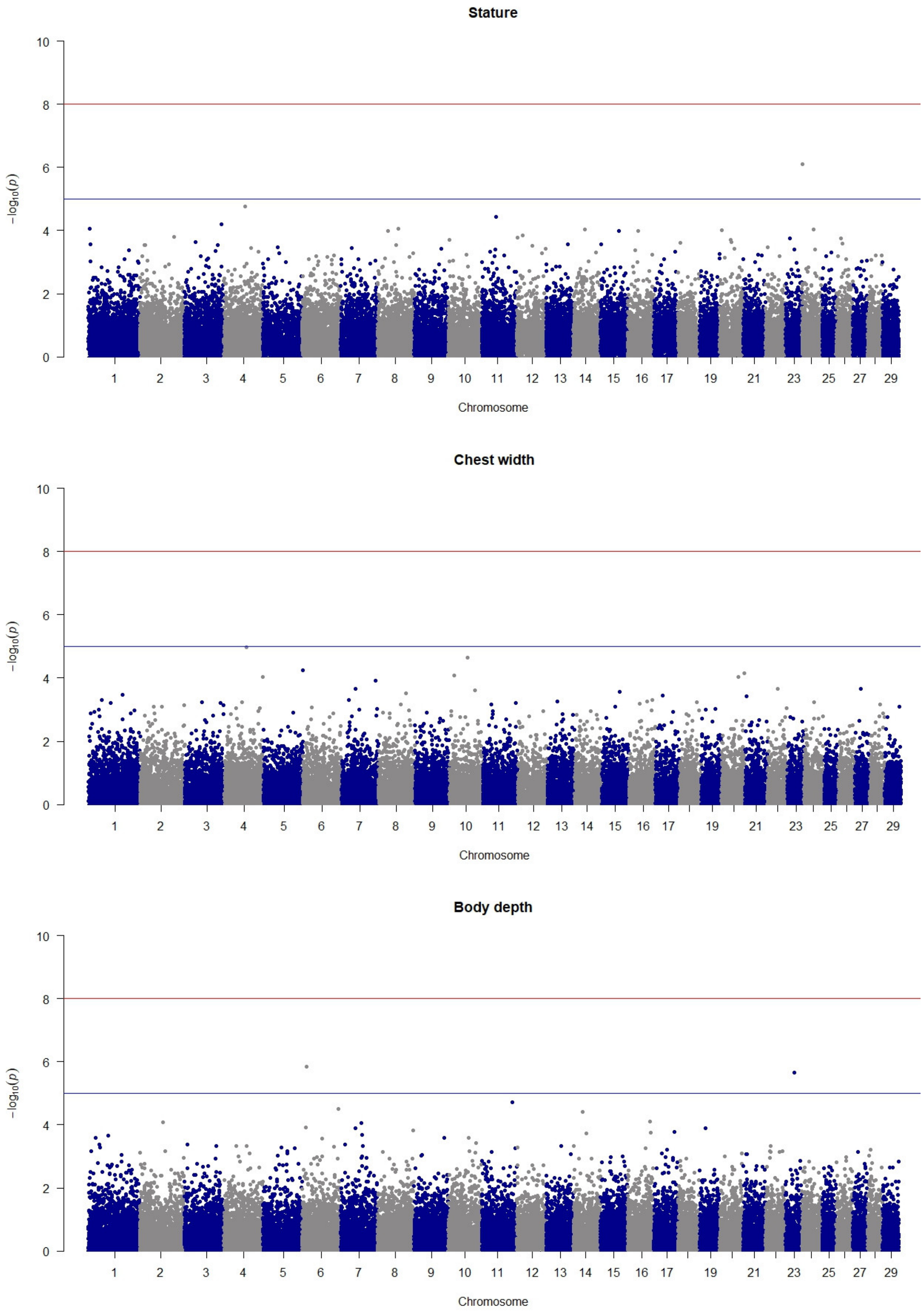
Stature
−log10(p)
Chromosome
Chest width
−log10(p)
Chromosome
Body depth
−log10(p)
Chromosome

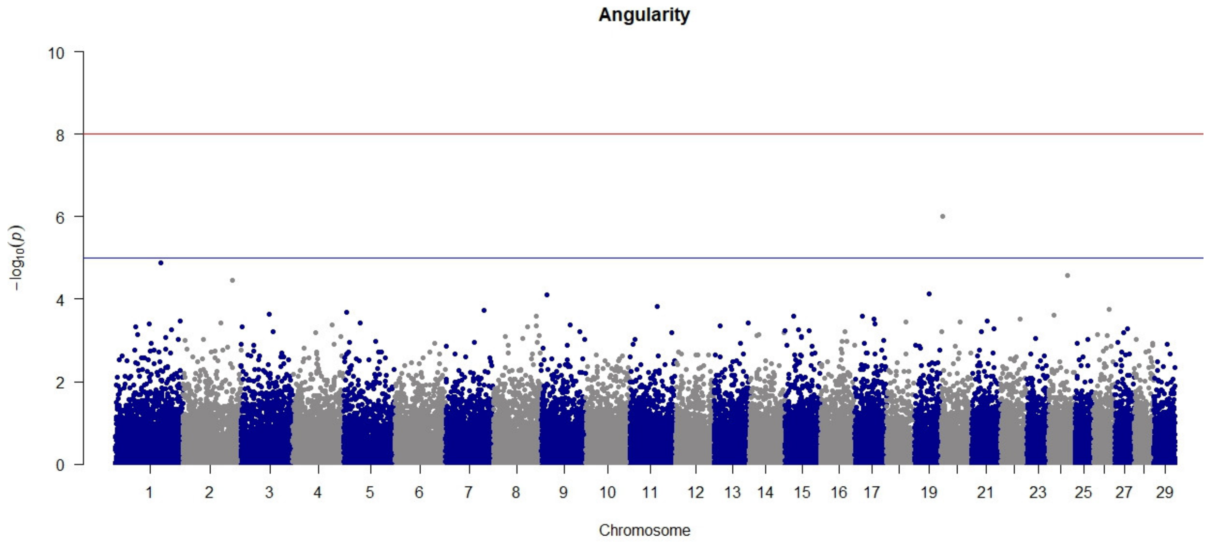
Angularity
10
8
6
4
2
0
$-\log_{10}(p)$
1 2 3 4 5 6 7 8 9 10 11 12 13 14 15 16 17 19 21 23 25 27 29
Chromosome

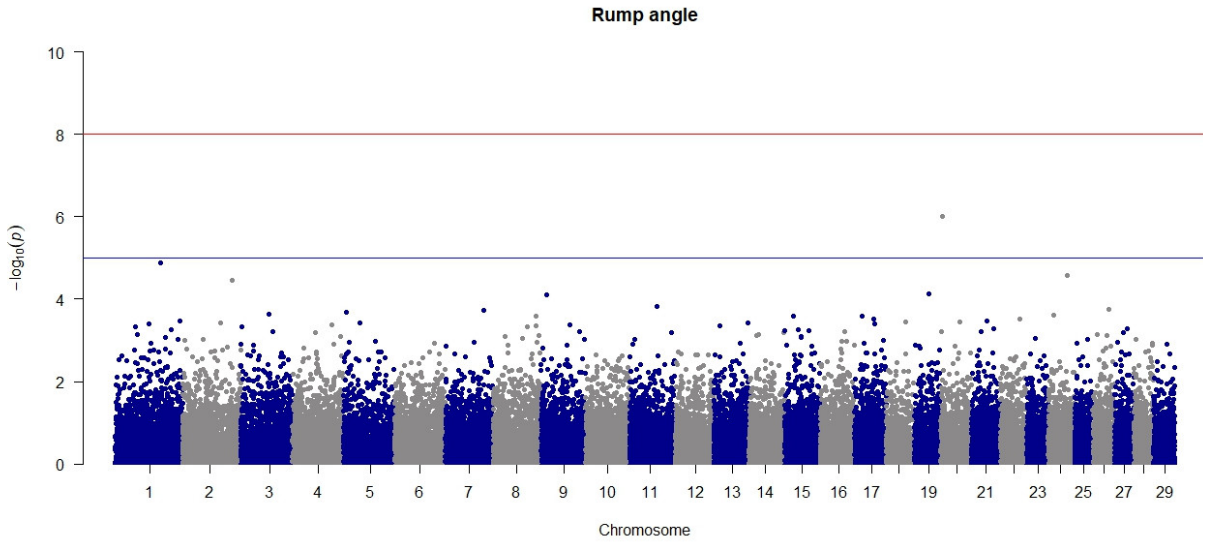
Rump angle
10
8
6
4
2
0
$-\log_{10}(p)$
1 2 3 4 5 6 7 8 9 10 11 12 13 14 15 16 17 19 21 23 25 27 29
Chromosome

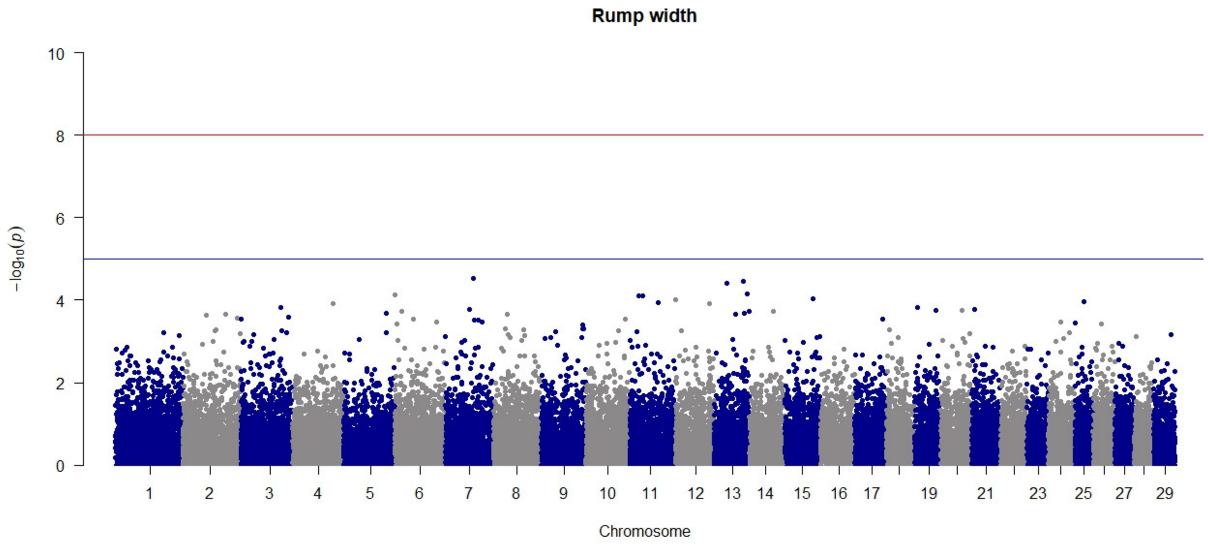
Rump width
10
8
6
4
2
0
$-\log_{10}(p)$
1 2 3 4 5 6 7 8 9 10 11 12 13 14 15 16 17 19 21 23 25 27 29
Chromosome

Rear legs (rear view)

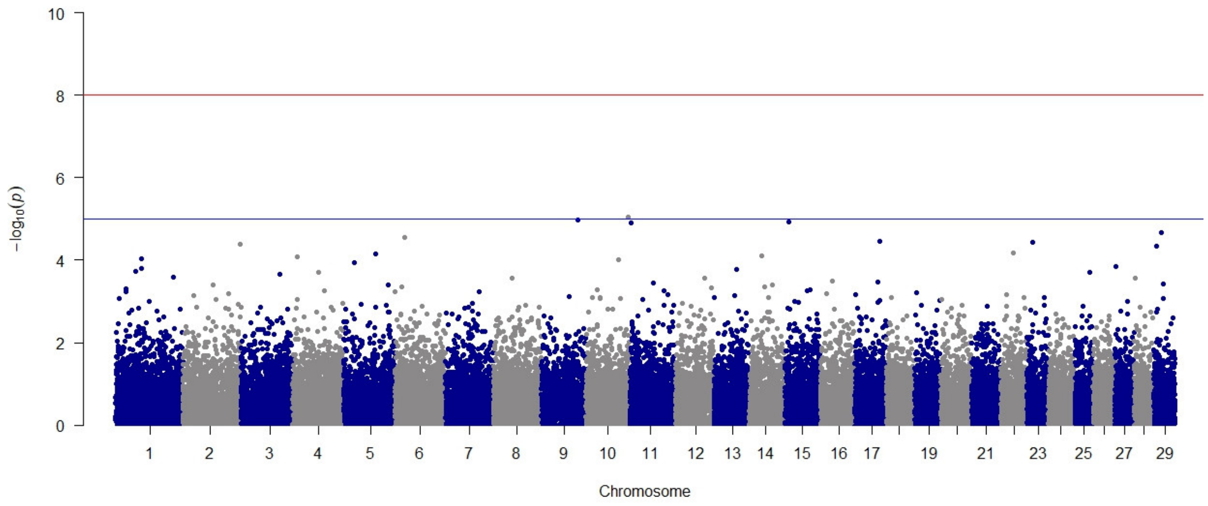

Rear legs (side view)

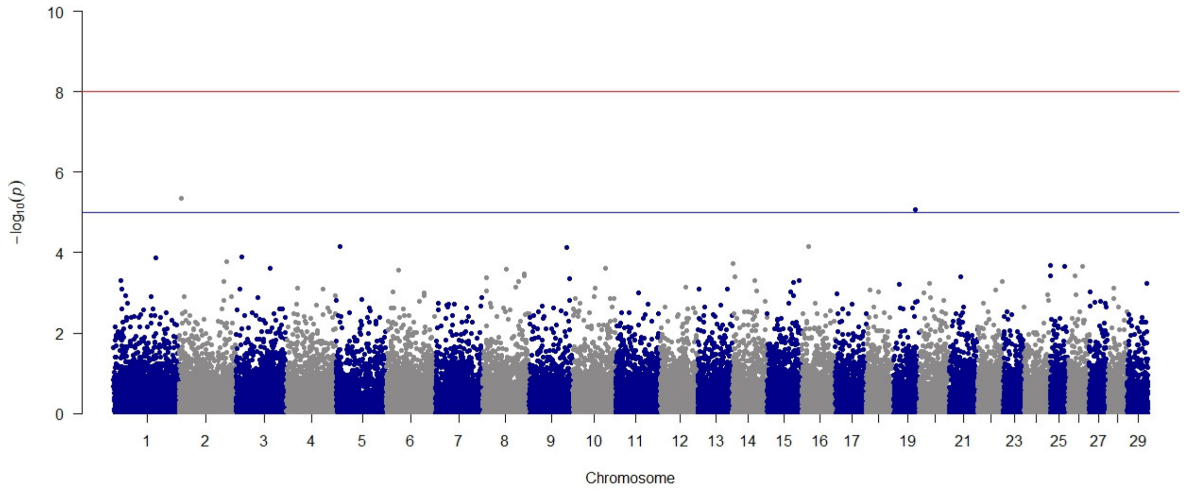

Foot angle

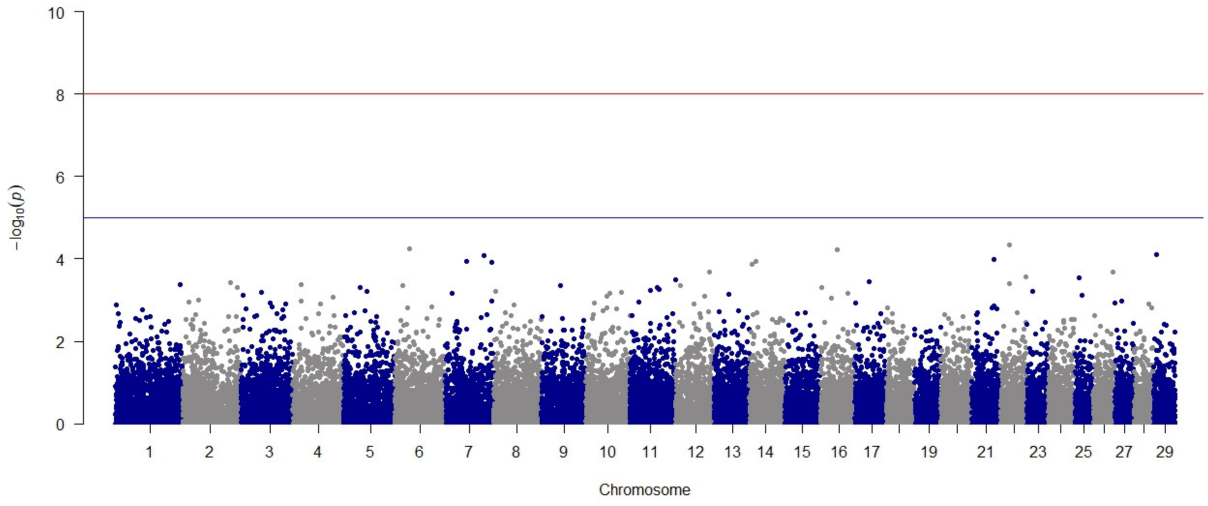

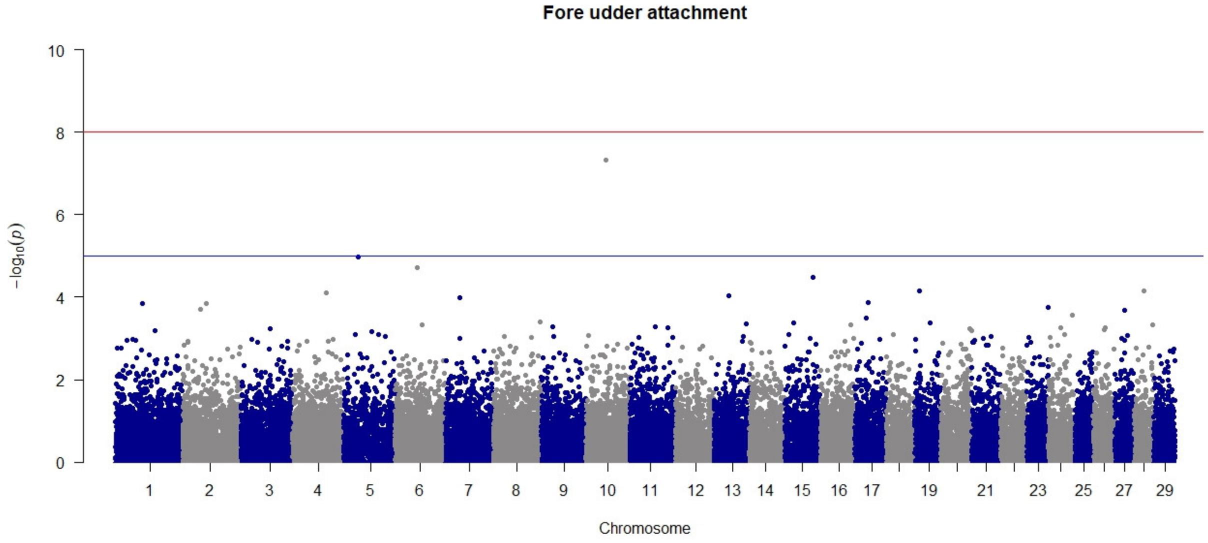
Fore udder attachment
10
8
6
4
2
0
−log10(p)
1 2 3 4 5 6 7 8 9 10 11 12 13 14 15 16 17 19 21 23 25 27 29
Chromosome

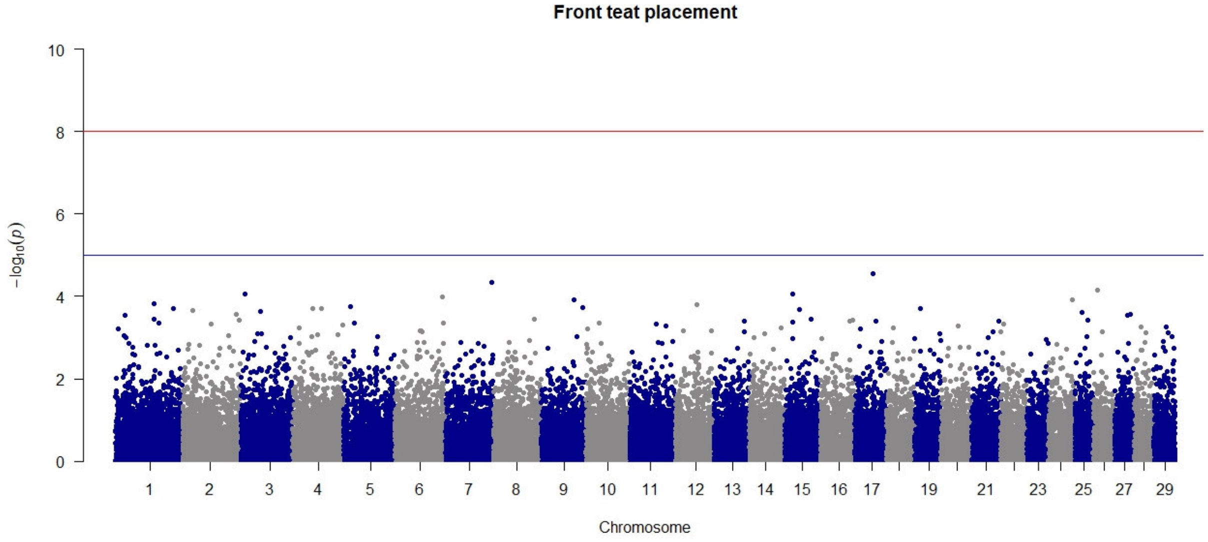
Front teat placement
10
8
6
4
2
0
−log10(p)
1 2 3 4 5 6 7 8 9 10 11 12 13 14 15 16 17 19 21 23 25 27 29
Chromosome

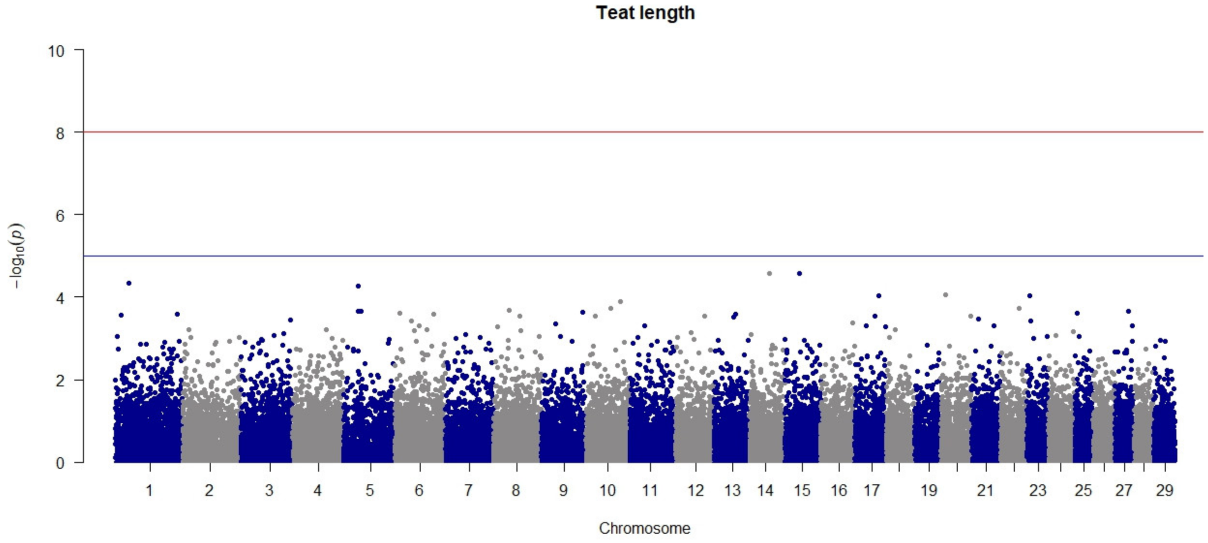
Teat length
10
8
6
4
2
0
−log10(p)
1 2 3 4 5 6 7 8 9 10 11 12 13 14 15 16 17 19 21 23 25 27 29
Chromosome

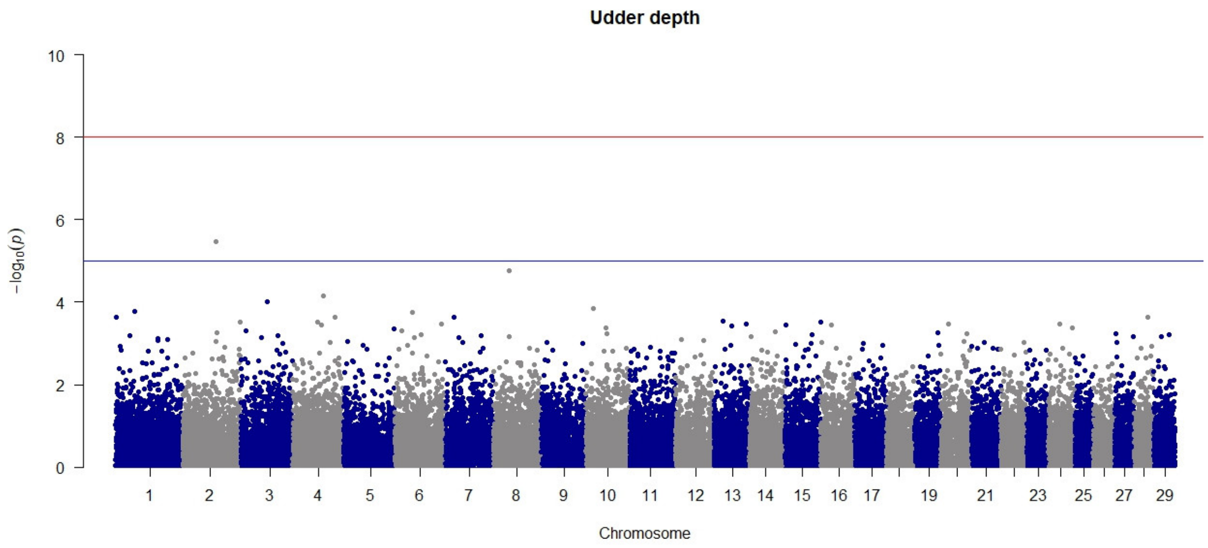
Udder depth
10
8
6
4
2
0
−log10(p)
1 2 3 4 5 6 7 8 9 10 11 12 13 14 15 16 17 19 21 23 25 27 29
Chromosome

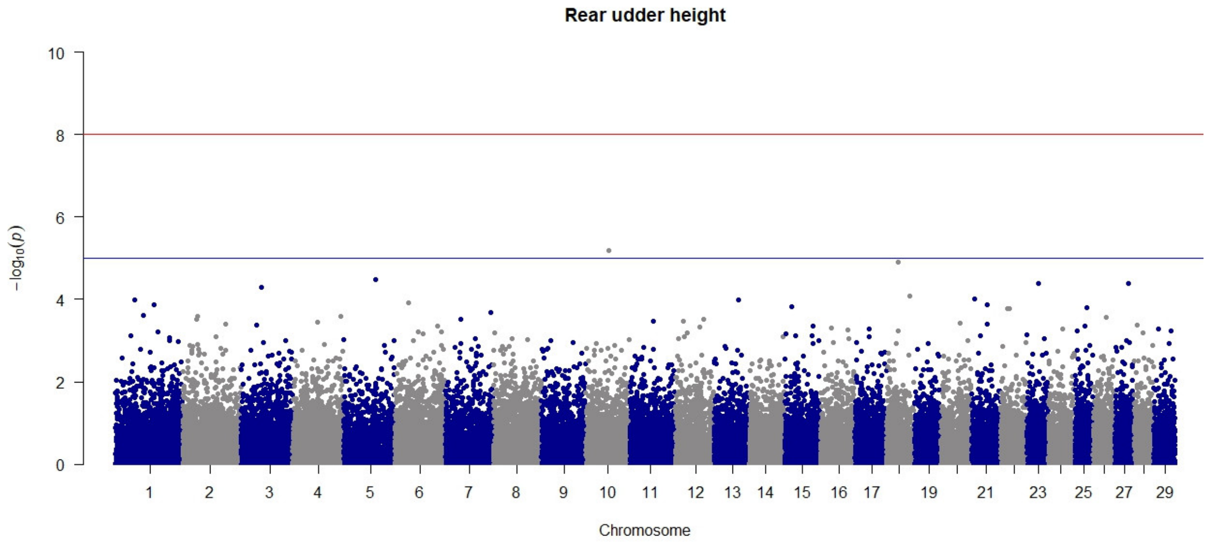
Rear udder height
10
8
6
4
2
0
−log10(p)
1 2 3 4 5 6 7 8 9 10 11 12 13 14 15 16 17 19 21 23 25 27 29
Chromosome

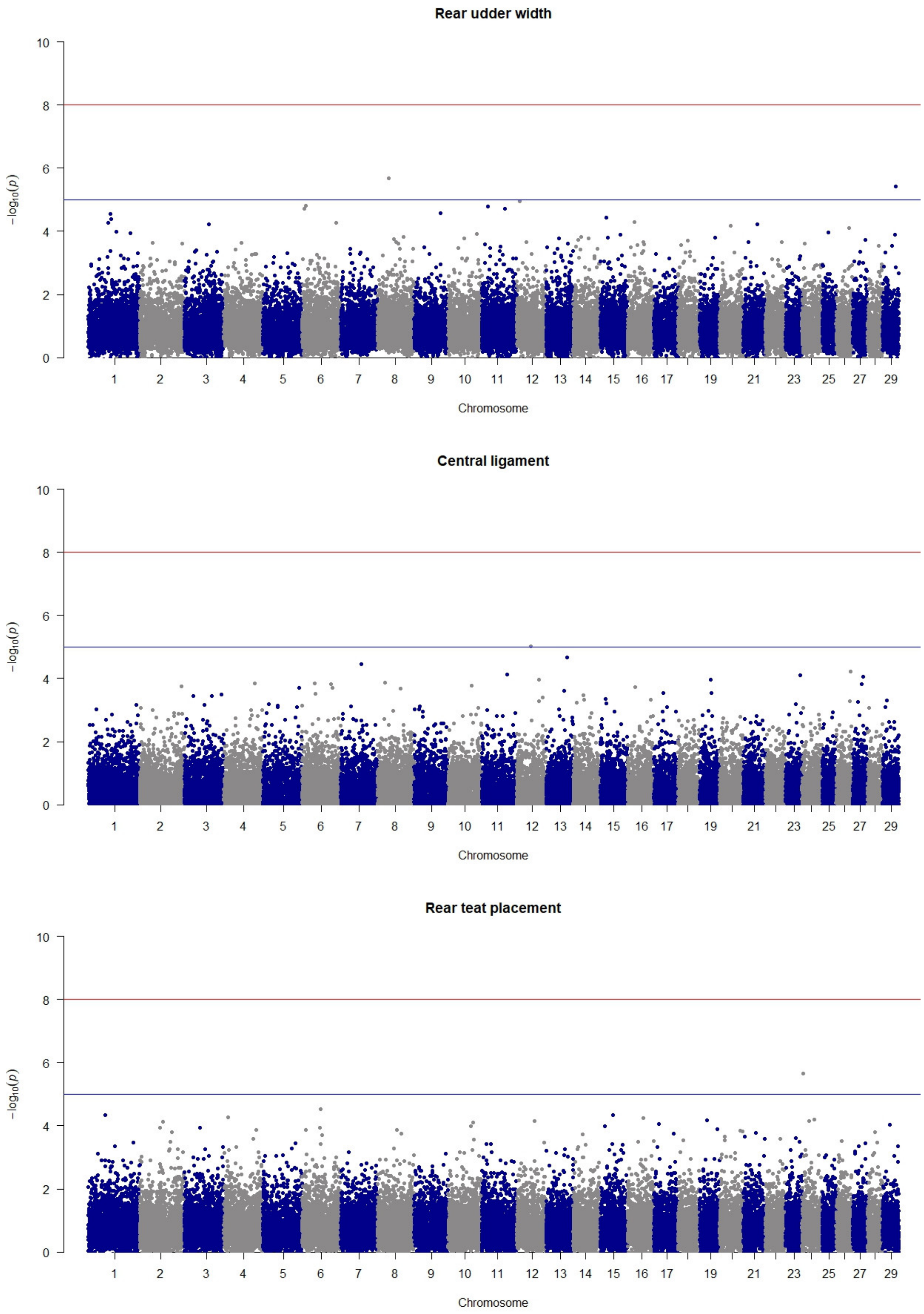
Rear udder width
10
8
6
4
2
0
−log10(p)
1
2
3
4
5
6
7
8
9
10
11
12
13
14
15
16
17
19
21
23
25
27
29
Chromosome
Central ligament
10
8
6
4
2
0
−log10(p)
1
2
3
4
5
6
7
8
9
10
11
12
13
14
15
16
17
19
21
23
25
27
29
Chromosome
Rear teat placement
10
8
6
4
2
0
−log10(p)
1
2
3
4
5
6
7
8
9
10
11
12
13
14
15
16
17
19
21
23
25
27
29
Chromosome

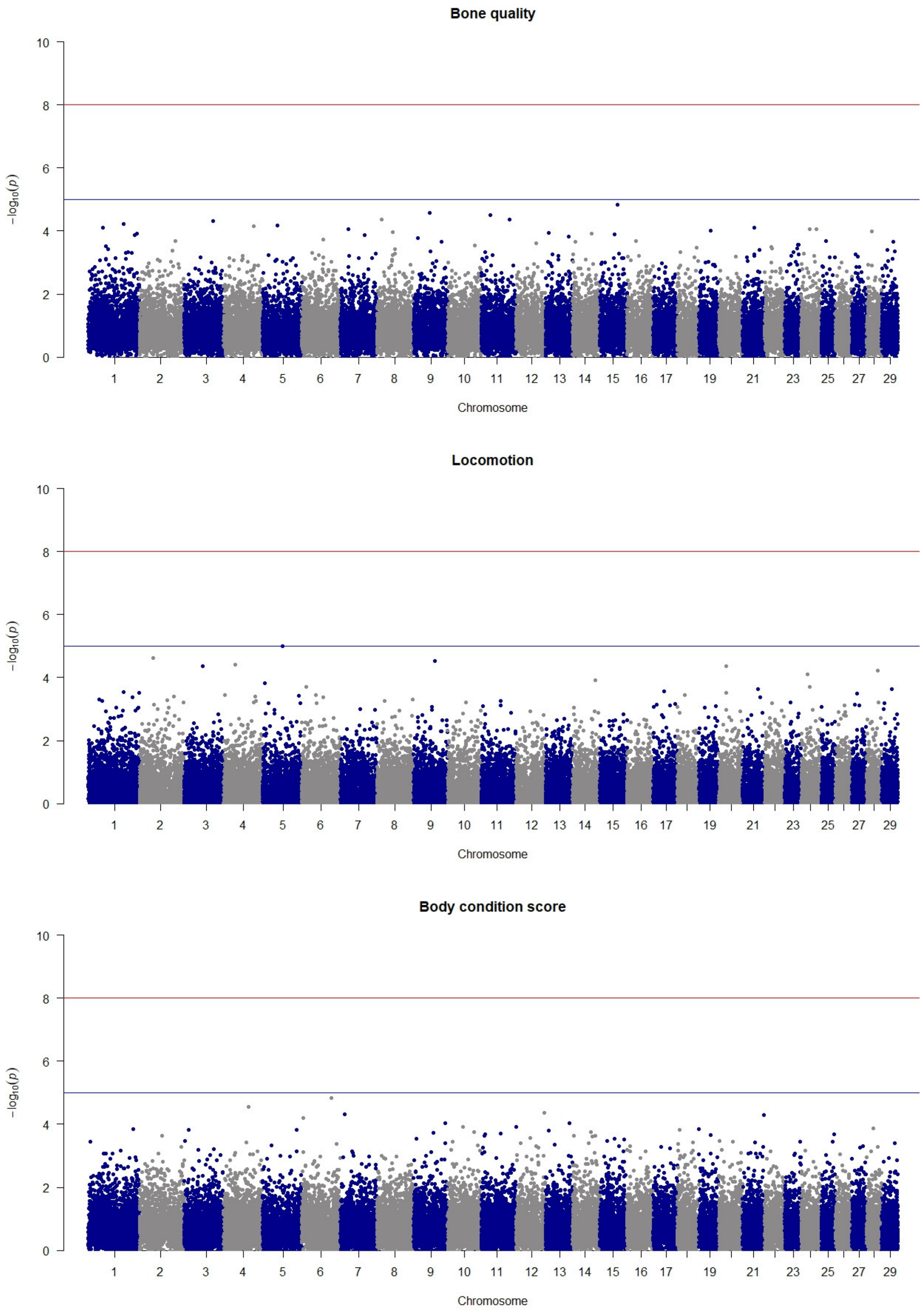
Bone quality
Locomotion
Body condition score
$-\log_{10}(p)$
0
2
4
6
8
10
1
2
3
4
5
6
7
8
9
10
11
12
13
14
15
16
17
19
21
23
25
27
29
Chromosome

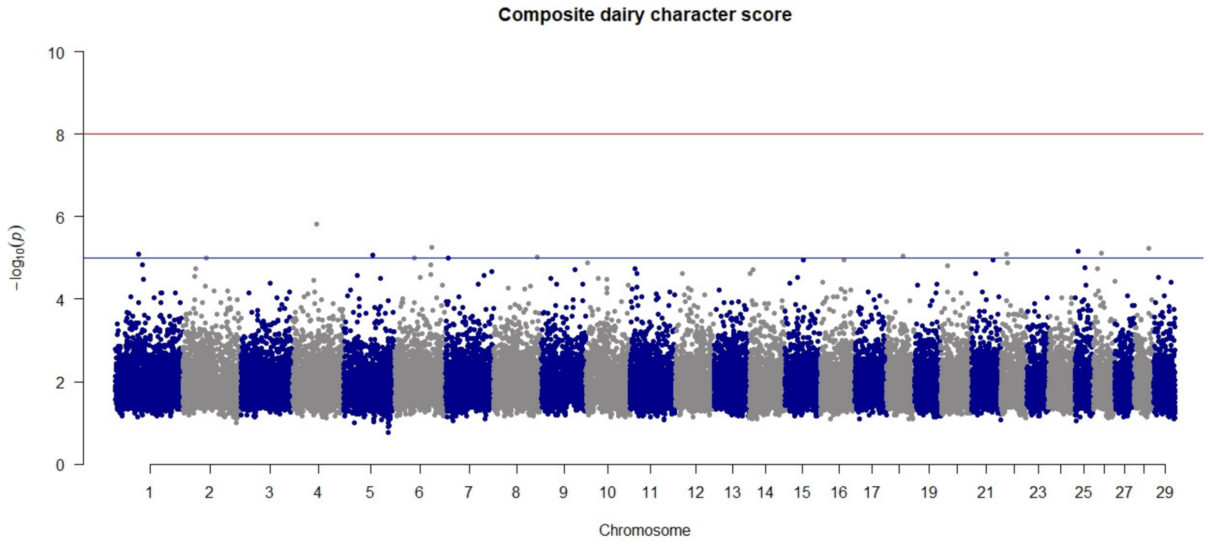
Composite dairy character score
−log10(p)
Chromosome

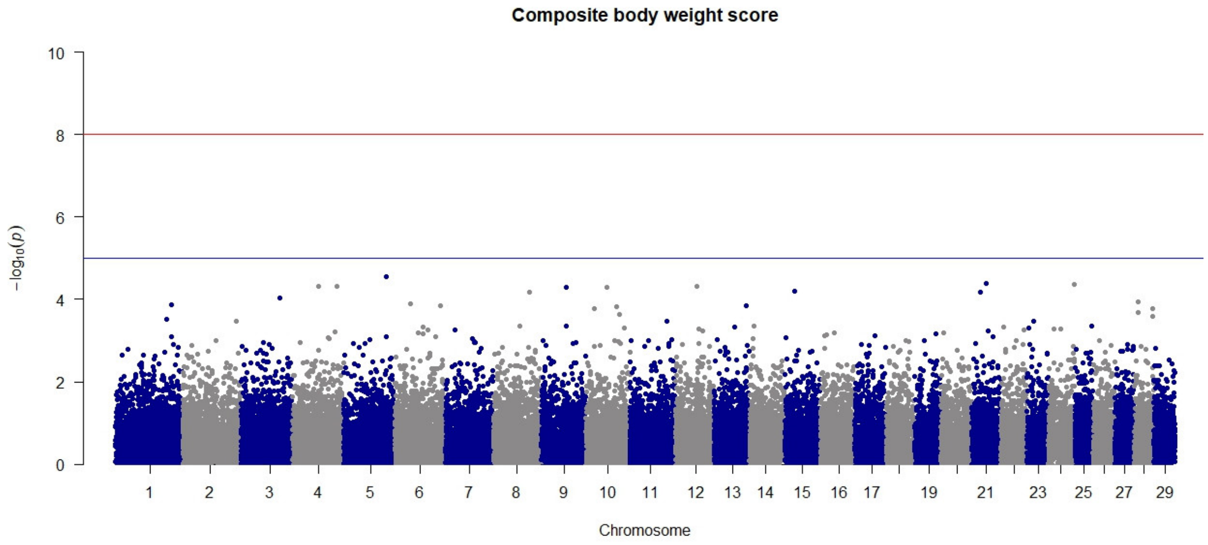
Composite body weight score
−log10(p)
Chromosome

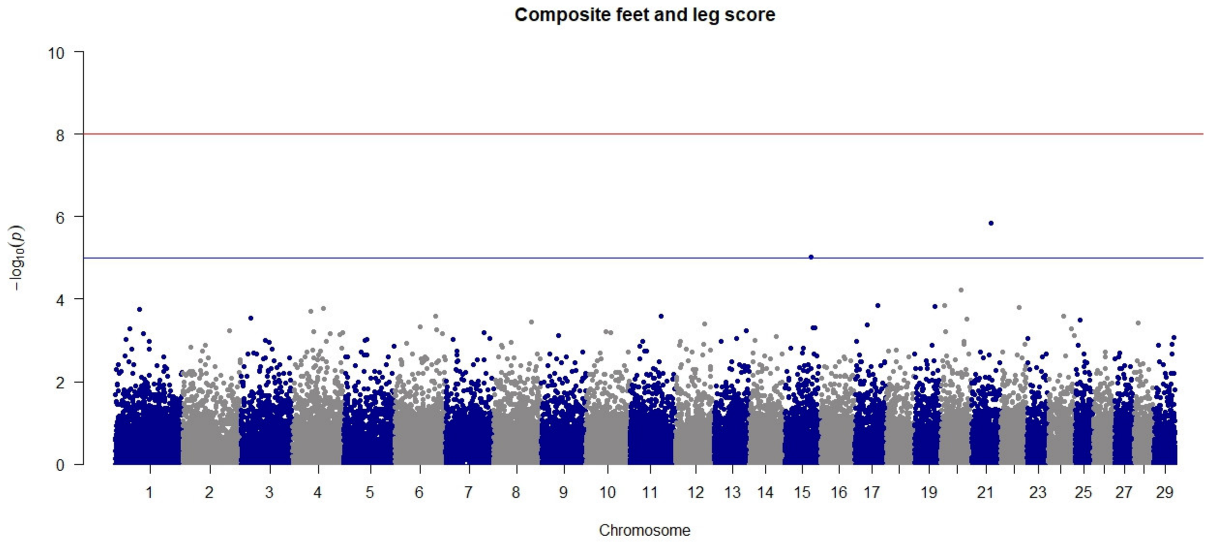
Composite feet and leg score
−log10(p)
Chromosome

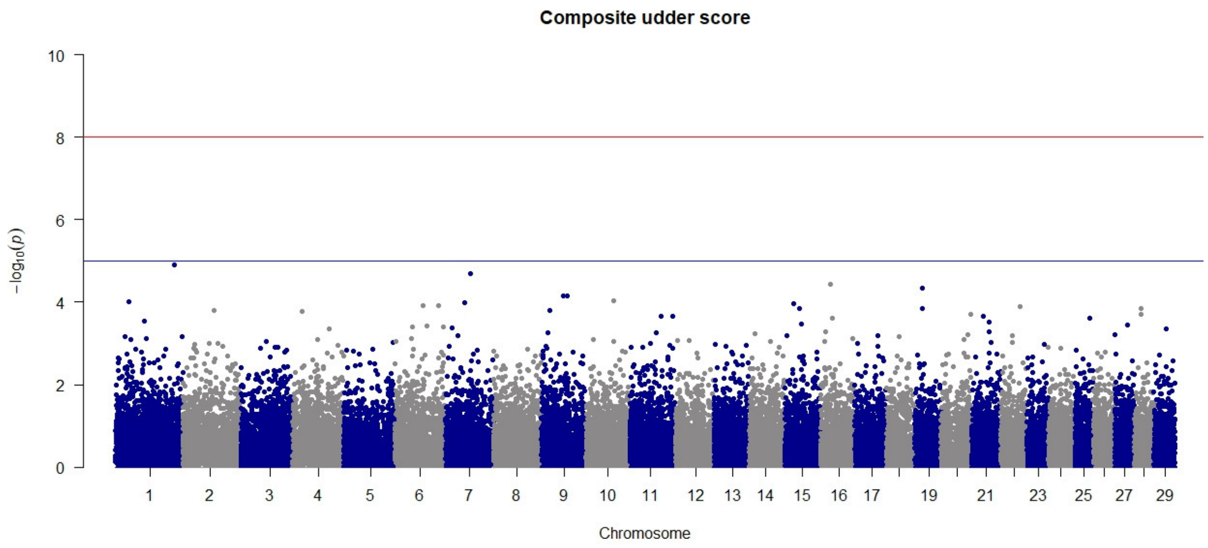

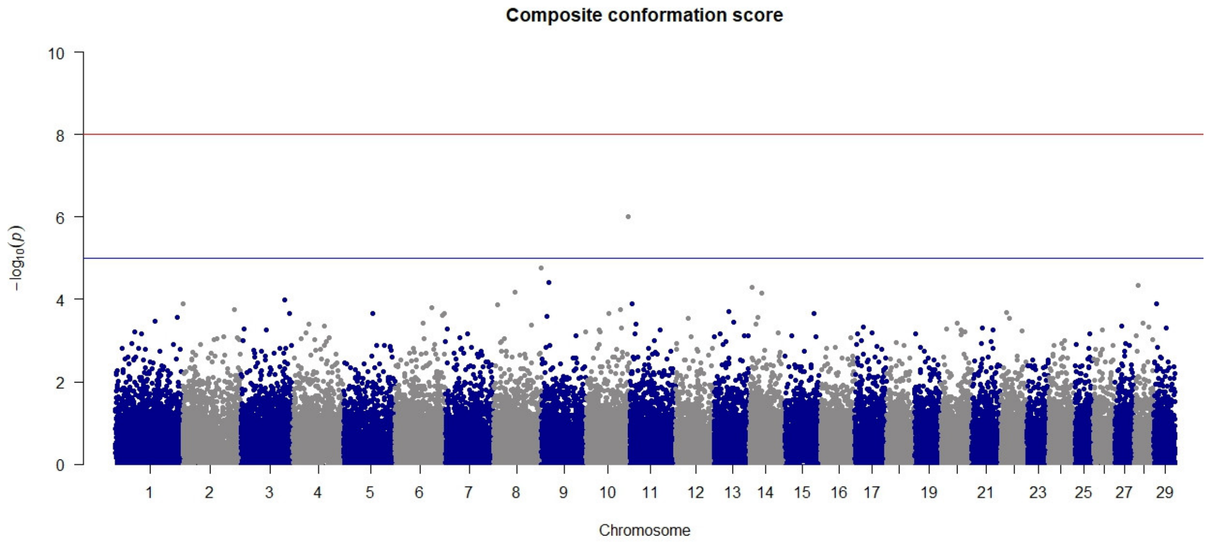

Figure S2. Manhattan plots of the GWAS analysis for 25 linear type traits in Czech Holstein. The level of significance was expressed by a p-value of 1.00×10^{-8} (genome-wide significance threshold, red line) and 1.00×10^{-5} (suggestive significance threshold, blue line).
